# Screening for celiac disease in 1st degree relatives: a 10-year follow-up study

**DOI:** 10.1186/1471-230X-14-36

**Published:** 2014-02-20

**Authors:** Rosa H Uenishi, Lenora Gandolfi, Lucas M Almeida, Patrícia M Fritsch, Fernanda C Almeida, Yanna K M Nóbrega, Riccardo Pratesi

**Affiliations:** 1 Graduate Program in Health Sciences, University of Brasilia School of Health Sciences, Brasilia, DF, Brazil; 2 Graduate Program in Medical Sciences, University of Brasilia School of Medicine, Brasilia, DF, Brazil; 3 Department of Pharmaceutical Sciences, University of Brasilia School of Health Sciences, Brasilia, DF, Brazil; 4 Research Center for Celiac Disease, University of Brasilia School of Medicine, Brasilia, DF, Brazil

**Keywords:** Celiac disease, First degree relatives, Serologic tests, Incidence, Prevalence

## Abstract

**Background:**

Although it is known that first degree relatives of celiac patients have an increased risk for celiac disease few studies are available on its incidence. We investigated the incidence of serologic conversion and of new cases of celiac disease among first degree relatives with negative results at a first screening.

**Methods:**

From a total of 634 first degree relatives of 186 biopsy-proven celiac disease patients diagnosed between October 2000 and October 2010, 450 subjects agreed to participate in the study (Group I), and underwent serologic screening. Between January 2010 and October 2012, out of the initial group of 450, 205 previously sero-negative subjects consented to participate in a second stage of the study and undergo new serologic testing (Group II). All serologically positive individuals of both groups (I and II) were genotyped for celiac disease-predisposing alleles (HLA-DQ2/DQ8).

**Results:**

19 subjects (4.2%) out of the 450 subjects of Group I disclosed positive serologic results, presence of DQ2 and/or DQ8 alleles and celiac disease-compatible mucosal abnormalities. The 205 previously negative first degree relatives from Group II that underwent new serologic testing disclosed eight sero-converted subjects. Mucosal abnormalities in five of these patients confirmed the diagnosis of celiac disease. During the 10-year period of the study the incidence of sero-conversion was 8/205 and the incidence of biopsy-proven celiac disease cases was 5/205.

**Conclusions:**

Our data are coincident with other works on this subject and confirm once again that relatives of celiac patients, especially first degree relatives are at high risk of developing celiac disease. In view of the relatively low incidence further studies are needed to try to establish a useful and cost-effective algorithm for follow-up of relatives of celiac patients.

## Background

Celiac disease (CD), an immune-mediated disorder of the gastrointestinal tract that eventually can affect several other organs and systems, may begin in either childhood or adult life. It is characterized by chronic inflammation of the small intestinal mucosa resulting in atrophy of intestinal villi, malabsorption, and a variety of clinical manifestations. During the last few decades has become progressively clear that CD is a frequent condition, although mainly due to the wide range of its clinical presentation a proportionally small number of cases are clinically recognized. It has been considered that CD affects 0.6 to 1.0% of the population worldwide [1,2], although these figures can vary widely depending on the world region under study.

CD was considered uncommon in developing countries until the 1990s, when the introduction of serologic screening tests resulted in increased rates of diagnosis in the Middle East, India, and North Africa [3], and Latin America [4]. An uneven prevalence for CD has been detected in different Brazilian regions, ranging from 1:119 to 1:417 in the general population [5,6] and from 1:214 to 1:681 in presumed healthy blood donors [7,8]. This is probably due to variances in environmental factors, dietary habits, and ethnic composition of the population.

CD has a strong genetic component and its genetic risk factors have been well characterized, as clearly suggested by higher prevalence among family members, and higher concordance rates in monozygotic twins than in dizygotic twins (83–86% vs 11%) [9]. The human leucocyte antigen (HLA) system is the major genetic determinant of CD predisposition, with the presence of specific HLA alleles accounting for approximately 40% of the disease susceptibility [10].

CD is a condition that can erupt at different life stages a first negative serologic test does not excludes the possibility of future onset of the disease. The problem of the incidence of new cases of CD among previously seronegative first degree relatives (FDRs) has been the subject of several papers during the last decade [11-15] including a recent study from the Southern region of Brazil [16]. Consequently, in the present study we investigated the incidence of serologic conversion and of new cases of CD among FDRs with negative serologic results at a first screening.

## Methods

### Study population and study design

From a total of 634 FDRs (parents, siblings and offspring) of 186 biopsy-proven CD patients (65 males, age range: 1 to 65 years, mean age: 13.0 ± 12.7; 121 females, age range: 1,1 to 62 years, mean age: 18.2 ± 14.7) who attended the Research Center for Celiac Disease of the Brasilia University Hospital and had been diagnosed between October 2000 and October 2010, 450 subjects [236 parents (99 fathers and 137 mothers, age range: 21 to 75 years, mean age: 45.9 ± 14.3), 147 siblings (63 brothers and 84 sisters, age range: 6 to 41 years, mean age 20.3 ± 11.7), 67 offspring (38 sons and 29 daughters, age range: 4 to 41 years, mean age: 13.5 ± 8.9)] could be contacted and agreed to participate in the present study. The Brasilia University Hospital is a public reference hospital that predominantly serves the low income population from the city of Brasilia and surrounding areas (Midwest Region of Brazil). These individuals usually depend exclusively on the Brazilian Unified National Health System. They exhibit mixed ancestry, with a significant contribution of Europeans intermixed with considerable input of other races, mainly Afro-descendants and Amerindians. All these 450 FDRs underwent serologic testing utilizing recombinant human transglutaminase antibody assay (IgA-htTG). All positive results on the tTG test were further confirmed by the antiendomysial antibody assay (IgA-EMA).

**Figure 1:**
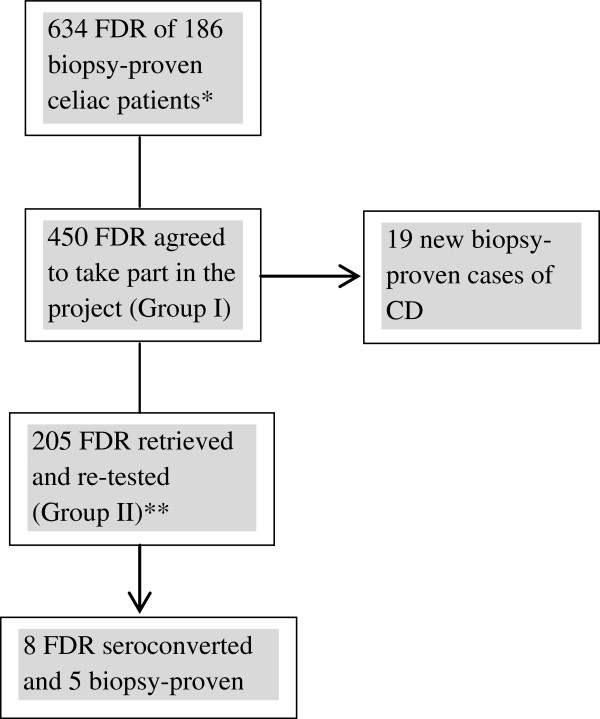
**Flow diagram of the study.** *These patients were tested between 2000 and 2010. **Research conducted between 2010 and 2012.

Between January 2010 and October 2012, out of the initial group of 450 individuals, 205 previously confirmed seronegative subjects [118 parents (44 fathers and 74 mothers, age range 21 to 75 years, mean age: 43.4 ± 10.2), 63 siblings (26 brothers and 37 sisters, age range: 6 to 47 years, mean age: 20.4 ± 11,9), 24 offspring (17 sons and 7 daughters, age range: 3 to 23 years, mean age: 13 ± 5.7)] consented to participate in the second stage of the study and undergo new serologic testing (IgA- htTG and IgA-EMA) (Group II). The first test of these 205 FDRs had been carried out at least a year earlier and all subjects were on a gluten-containing diet at the time of the second test. Intestinal biopsy was suggested to all patients that disclosed at least one positive test. The flow diagram of the study can be seen in Figure 1.

### Serologic markers

All subjects were previously tested for serum immunoglobulin IgA. The initial serologic screening was performed utilizing the ELISA IgA antitransglutaminase antibody assay (QUANTA Lite® htTG IgA ELISA, INOVA Diagnostic Inc., San Diego, CA, U.S.A.). All positive results, determined according to the manufacturer’s recommendation values, were further confirmed by the IgA-EMA assay tested by indirect immunofluorescence with two independent observers examining all slides.

Briefly, sera were placed onto 4 μm thick cryosections of distal monkey oesophagus (INOVA Diagnostic Inc., San Diego, CA, U.S.A.), fixed to slides at a dilution of 1:5 with phosphate buffered saline and incubated for 30 min. The final reaction was detected with fluorescein isothiocyanate (FITC) rabbit anti-human IgA conjugate (Biosystems, Barcelona, Spain). The result was considered positive when bright green fluorescence of the reticulin-like staining of smooth muscle was seen.

### HLA typing

To further support the diagnosis of CD all serologically positive individuals from both groups (group I and group II) were genotyped for the presence of HLA predisposing alleles for CD. Briefly, genomic DNA was extracted from peripheral venous blood samples using the IllustraTMBlood genomicPrep Mini Spin Kit (GE Healthcare, Buckinghamshire, UK). HLA-DQA1*0501 (DQ2 α chain), HLA-DQB1*02 (DQ2 β chain), HLA-DQA1*0301 (DQ8 α chain) and DQB1*0302 (DQ8 β chain) genotyping was performed by polymerase chain reaction amplification using sequencespecific primers (PCR-SSP). For internal positive amplification control, each PCR reaction included a primer pair for a conserved region of the DRB1 gene. The amplified products were separated using 2% agarose gel, stained with ethidium bromide and then visualized under a ultraviolet (UV) trans illuminator.

### Ethical issues

The study complies with the principles of the latest Declaration of Helsinki (2008) and was approved by the Ethics Committee of the Brasilia University School of Health Sciences. All eligible patients were informed about the objectives of the research and the potential necessity of small intestine biopsy. Informed consent was obtained from adults and from the parents for their children. Additional consent for publication of their clinical data was obtained from the patients listed in the Table 1.

**Table 1:** Clinical and laboratory data of eight CD patients originating from the initial group of 450 FDR that disclosed seroconversion (Group II)

^**$**^**Pts**	**Kinship**	**Age at 1**^**st **^**test (y)**	**Age at 2**^**nd **^**test (y)**	**Interval between 1**^**st **^**and 2**^**nd **^**test (m)**	**tTG**	**EMA**	**HLA**	^**#**^**Marsh**	**Symptoms and associated disorders**
1	Sister	6	7	12	35.9	Pos	DQA1*0501	I	Irritability, constipation, recurrent abdominal pain
							DQB1*0201		
2	Sister (twin)	12	16	54	35.1	Neg	DQA1*0501	ND	Recurrent abdominal pain, frequent joint pain
							DQB1*0201		
							DQA1*0301		
							DQB1*0302		
3	Sister	30	37	88	123.6	Pos	DQA1*0501	III	Bouts of diarrhea, recurrent abdominal pain, bloating, anemia
							DQB1*0201		
4	Mother	30	37	84	46.5	Neg	DQA1*0501	ND	Bouts of diarrhea, aphthous ulcers, bloating, joint pain
							DQB1*0201		
5	Sister	32	35	38	57,1	Neg	DQA1*0501	ND	Asymptomatic
							DQB1*0201		
6	Sister	37	39	26	81.7	Pos	DQA1*0501	I	Bloating, constipation, aphtous ulcers, fatigue
							DQB1*0201		
7	Sister and daughter	37	44	89	95.6	Pos	DQB1*0201	III	Asymptomatic
							DQA1*0301		
							DQB1*0302		
8	Father	67	75	89	65.9	Pos	DQA1*0501	II	Asymptomatic
							DQB1*0201		

^$^Patients ^#^Marsh Classification.

## Results

No cases of IgA deficiency could be detected among the study subjects. The search for IgA-htTG antibodies among the initial 450 FDRs (Group I) yielded 19 positive results on tTG assay, that were further confirmed by the IgA-EMA immunofluorescent test. Additionally, HLA typing revealed the presence of DQ2 and/or DQ8 predisposing alleles in all these sero-positive subjects. Intestinal biopsy was suggested to these patients and small bowel histologic evaluation confirmed the diagnosis of CD. Consequently, the prevalence of CD among the 450 FDRs of 186 celiac patients was 19/450 (4.2%).

The 205 FDRs of Group II that underwent a new serologic test disclosed eight previously sero-negative FDRs that had experienced sero-conversion. Five of these newly seroconverted subjects underwent a duodenal biopsy that showed variable degrees of mucosal abnormalities, confirming their diagnosis of celiac disease. The characteristics of this second group can be seen in Table 1. The mean interval of time between the first and the second test was 51.1 months ± 28.7. During the 10-year period of the study the incidence of sero-conversion was 8/205 and the incidence of biopsy- proven CD cases was 5/205.

The incidence rate of sero-conversion in our FDRs during a period of 10 years was 8/205, corresponding to an annual incidence of 0.141 (95% - CI: 0.7 - 1.0). Among the eight FDRs the diagnosis of CD was confirmed in five (5/205) which corresponds to an annual incidence of 0.098 (95% - CI: 0.7 - 1.0).

## Discussion

CD is a common disorder, with a prevalence of nearly 1% in Western populations [17,18]. In countries of high prevalence a minority of authors maintain the view that the general population should undergo systematic screening for this disorder because it fulfills the major criteria for mass screening [19,20]. Other authors argue against mass screening pointing to the still incomplete understanding of the natural course of the disease, poor adherence to a gluten-free diet, conflicting mortality rate regarding undiagnosed CD and lack of data regarding cost-effectiveness [21,22]. Additionally, studies in adults have identified individuals who were serologically negative at a first screening, and later underwent sero-conversion, disclosing villous atrophy on duodenal biopsy [23]. Consequently, at the present time the general consensus is that there are not enough data to justify screening for CD in the general population [24]. On the other hand, in at risk groups, such as patients with type 1 diabetes, autoimmune thyroid and liver diseases and, as the single most important risk factor, a family history of biopsy-proven CD, screening for CD is recommendable and would probably have a favorable cost-benefit ratio [21].

HLA typing for DQ2/DQ8 alleles has been suggested as a second step in previously serologically negative FDRs to limit the number of subjects for serologic follow-up, since HLA typing is an efficient tool to discriminate individuals who will regularly require clinical and serological control [10,25,26]. The negative predictive value of HLA typing is high due to the fact that gluten intolerance rarely occurs in the absence of HLA-predisposing alleles [27]. Although several PCR kits for HLA sampling are currently available at a relatively lower cost, their use is not yet covered by the Brazilian Unified National Health System, and this fact drastically reduces its practicality in extensive screening programs in our country.

Studies on prevalence of CD among FDRs of celiac patients have shown significant variability, probably secondary to differences on methods of diagnosis (biopsy vs. serologic testing) and country of study. A systematic review performed by Dubé et al. [28] disclosed an estimated prevalence of CD in FDRs undergoing intestinal biopsy of 16%. On the other hand, considering that CD is a dynamic process and the risk of developing the disease is spread over time, it is expected that FDRs will show not only an increased prevalence but also an increased incidence of the disorder. Several studies have focused on this topic and it has been estimated that between 1.6 and 6.6% of previously serologically negative FDRs, may seroconvert within a range of 0.5 to 20 years resulting in an annual incidence ranging from 0.3 to 1.7 [26].

In our study 45.5% of the FDRs (205/450) could be retrieved during the follow-up period. Eight patients showed increased level of IgA-htTG. Three of these eight patients were IgA-EMA negative and consequently a definitive diagnosis of CD was not established; one was asymptomatic and the other two disclosed complaints compatible with CD. The mother of the patient N°2, considered of high risk for CD, preferred to start the child on GFD, omitting the biopsy procedure. The other two patients also denied consent for biopsy, preferring to wait for further testing. All these three patients were re-scheduled for future follow-up.

The data shown in Table 1 reinforce the known fact that an increased frequency of gluten sensitivity is predominantly found among females and among siblings. Also of interest is that two of the new cases (patients 7 and 8) were members of the same Family of 16 in which, out of the 11 members that could be retested, five were biopsy-proven celiac, 10 were carriers of predisposing HLA alleles and only one was serologically and DQ2/DQ8 negative. This confirms the known fact that members of families with multiple cases are at higher risk of developing the disease [29].

The incidence of 3.9% of sero-conversion found in our study, in a period of 10 years is similar to the values found in previous published studies [11-15]. Comparing with our findings, the increased rate of sero-conversion found by Nass et al. [16] in the Southern region of Brazil is probably due to the predominantly European origin of the population screened, which again highlights the heterogeneity of the Brazilian racial composition.

## Conclusion

Our data are in line with other works on this subject and confirm once again that relatives of celiac patients, especially FDRs are at high risk of developing CD. To reach the best strategy the true incidence in the various age groups still need further and more powerful studies [30]. To establish an effective algorithm to be applied in our country is still more difficult in view to the low educational and economic level of the population that attends our hospital and is mainly covered by a Governmental Unified Health System that still do not consider CD as a potential public health problem.

Presently, in our department we are performing HLA typing in all the FDRs and repeating the serologic testing every two years or previously, depending on the appearance of suggestive CD symptomatology although we know that this is hardly a strategy that could be generalized to other regions of this country.

## Competing interests

The authors declare that they have no competing interests.

## Authors’ contribution

RHU and LG drafted the manuscript. LMA carried out the molecular genetic studies. FCA and PMF carried out the immunoassays. LG conceived the study. RP and YKMN participated in the design, coordination of the study. RP and YKMN helped to draft the final version of the manuscript. All authors read and approved the final manuscript.

## Pre-publication history

The pre-publication history for this paper can be accessed here:

http://www.biomedcentral.com/1471-230X/14/36/prepub
